# The facultative human oral pathogen *Prevotella histicola* in equine cheek tooth apical/ periapical infection: a case report

**DOI:** 10.1186/s12917-021-03048-9

**Published:** 2021-10-30

**Authors:** Silvio Kau, Michael D. Mansfeld, Alexandra Šoba, Timo Zwick, Carsten Staszyk

**Affiliations:** 1 Institute of Morphology, Working Group Anatomy, Department of Pathobiology, University of Veterinary Medicine Vienna, Vienna, Austria; 2 Carinthian Institute of Veterinary Disease Control, Klagenfurt, Austria; 3 Synlab-vet, Augsburg Laboratory, Augsburg, Germany; 4 Department of Equine Dentistry and Maxillofacial Surgery, Veterinary Clinic Gessertshausen, Gessertshausen, Germany; 5 Institute of Veterinary Anatomy, Histology and Embryology, Faculty of Veterinary Medicine, Justus-Liebig-University Giessen, Giessen, Germany

**Keywords:** Horse, Nasal discharge, Dental infection, Infundibular caries, Odontogenic sinusitis, Conchal necrosis, Exodontia, Oral flora, Oral pathogen

## Abstract

**Background:**

*Prevotella histicola* is a facultative oral pathogen that under certain conditions causes pathologies such as caries and periodontitis in humans. *Prevotella spp*. also colonize the oral cavity of horses and can cause disease, but *P. histicola* has not yet been identified.

**Case presentation:**

A 12-year-old Tinker mare was referred to the clinic for persistent, malodorous purulent nasal discharge and quidding. Conservative antibiotic (penicillin), antiphlogistic (meloxicam), and mucolytic (dembrexine-hydrochloride) treatment prior to referral was unsuccessful and symptoms worsened. Oral examination, radiography, sino-/ rhinoscopy, and standing computed tomography revealed severe apical/ periapical infection of the upper cheek tooth 209 with accompanying unilateral sinonasal inflammation and conchal necrosis. The tooth exhibited extensive subocclusal mesial infundibular cemental hypoplasia and caries, and an occlusal fissure fracture. After mechanical debridement and thermoplastic resin filling of the spacious subocclusal carious infundibular lesion, the tooth was extracted intraorally. The sinusitis and conchal necrosis were treated transendoscopically. Selective bacteriological swab cultures of affected tooth roots and subsequent matrix-assisted laser desorption ionization-time of flight mass spectrometry showed an infection with the obligate anaerobic, Gram-negative bacterium *P. histicola*. Surgical intervention and adapted antibiotic therapy led to normal healing without complications.

**Conclusions:**

This study provides the first documented case of dental infection in a horse caused by *P. histicola* at once indicating necessity of more sufficient microbiological diagnostics and targeted antibiotic treatment in equine dental practice. This finding is also conducive to understand species-specific *Prevotella* diversity and cross-species distribution.

## Background

Apical infection of upper cheek teeth and associated alveolar ostitis are a well-known cause of secondary sinonasal disorders in the horse [1–6]. The last premolar to second molar teeth (08 – 10) were found most frequently involved in dental sinusitis cases [1, 6–8]. Dentogenic sinonasal disorders are often accompanied by purulent malodourous nasal discharge [1, 9], which is most common with infections in the maxillary molars [2].

In horses, primary apical affections are suggested to be of bacterial origin [2] whereas no bacteria genera were found being exclusively related with a distinct type of cheek tooth pathology [10]. Anachoretic pulpitis is likely among the most important reasons causing apical infection in the horse [11, 12]. Bacterial pulpitis and apical/ periapical infection may also occur due to fractures or fissure fractures of the clinical crown and therefore occurring primary pulp exposure and bacterial invasion, due to periodontal spread of bacteria, dysplasia, or extension of infundibular caries [11–15].

First described in 2008, *Prevotella histicola*, a strict anaerobe, Gram-negative-staining, rod-shaped bacterium has been identified as an oral commensal in humans [16, 17], which under certain conditions can overcome cellular barriers inducing various orodental pathologies such as periodontitis or caries [16, 18–21]. *Prevotella* ssp. - other than *P. histicola* or solely on the genus level - were previously found in the horse as part of the normal accompanying flora and in connection with various pathological conditions at distinct body sites. In horses, *Prevotella* ssp. (former *Bacteroides*) have been isolated from healthy oral/ gingival tissues and both dental and secondary sinus disease, e.g. periodontitis, pulpitis, apical infection, and dental sinusitis, but were not further sub-characterized [10, 22–26]. In addition to *Actinomyces* ssp., *Actinobacillus* ssp., *Fusobacterium* ssp., *Leptotrichia* ssp., *Porphyromonas* ssp., *Streptococcus* ssp., or *Veillonella* ssp., *Prevotella* ssp. have been most commonly identified with exodontia-associated bacteremia in horses [10, 26]. In horses, *Prevotella* ssp. on the genus level were also found in healthy pharyngeal tonsillar tissue, oesophageal tissue, gastral tissue, and in septic sialoadenitis [27–30]. *Prevotella dentasini*, isolated from oral tissues in donkeys, was previously described as a novel species by Takada *et al*. (2010) [31]. Just recently, *P. melaninogenica* and *P. oralis* have been isolated from an aspirate of a purulent medial pterygoid muscle myositis, presumably of oral origin, in a horse [32]. Santoshi and Leshem (2014) isolated *P. melaninogenica* from a horse bite wound in a human patient [33].

Both in humans and horses the complexity of the oral microbiome and associated requirements for survival and pathogenicity of microorganisms remain incompletely clarified. The genus *Prevotella* exhibits a high diversity with over 50 detected phylotypes [16, 34], however, little is known about the species diversity of the genus *Prevotella* and cross-species spread between humans and various animal species in health and disease. The current study describes and illustrates a complex case of a first documented dental infection with *P. histicola* and an extensive accompanying sinonasal affection. It further highlights the urgent need of sufficient microbiological examination and well-directed antibiotic treatment as this should be state of the art in equine dental practice.

## Case presentation

### Case history

A 12-year-old Tinker mare, showing a body weight of 605 kg and body conditioning score or six, was referred to the Department of Equine Dentistry and Maxillofacial Surgery, Veterinary Clinic Gessertshausen, Germany. The horse showed continuous unilateral malodorous purulent nasal discharge from the left nostril and quidding during one week before referral. Prior to referral the horse already received antibiotic treatment (penicillin), antiphlogistic drugs (meloxicam) and expectorative mucolytic therapy (dembrexine hydrochloride) for a period of one week. This conservative treatment attempt was unsuccessful and symptoms worsened. The last dental prophylaxis was carried out by the private veterinarian approximately one year ago. Contemporary confirmation for active immunization against tetanus was present and the horse was officially not intended for human consumption.

### Clinical findings

The horse showed a normal general condition, heart rate of 36 bpm and breathing rate of 14/ minute. Mucous membrane color and capillary refill time were normal at presentation. The rectal temperature of 37.9 °C was within the normal range. Auscultation of the heart, lungs and gastrointestinal tract revealed no abnormalities. The horse showed a persistent unilateral, moderate to high grade, carious-smelling purulent nasal discharge from the left nostril and no respiratory stridor. The left mandibular lymph nodes were slightly enlarged. Craniofacial bones and soft tissues exhibited no asymmetries. Fundoscopy and eye reflexes revealed no changes on both sides; both serve as a proof of pre-operative *status chirurgicus* and thus may help to avert any erroneous claim of indemnification in case of complications due to treatment, e.g. maxillary nerve block which is adjacent to sensible eye structures.

### Primary and diagnostic findings

#### Orodental examination

**Fig. 1:**
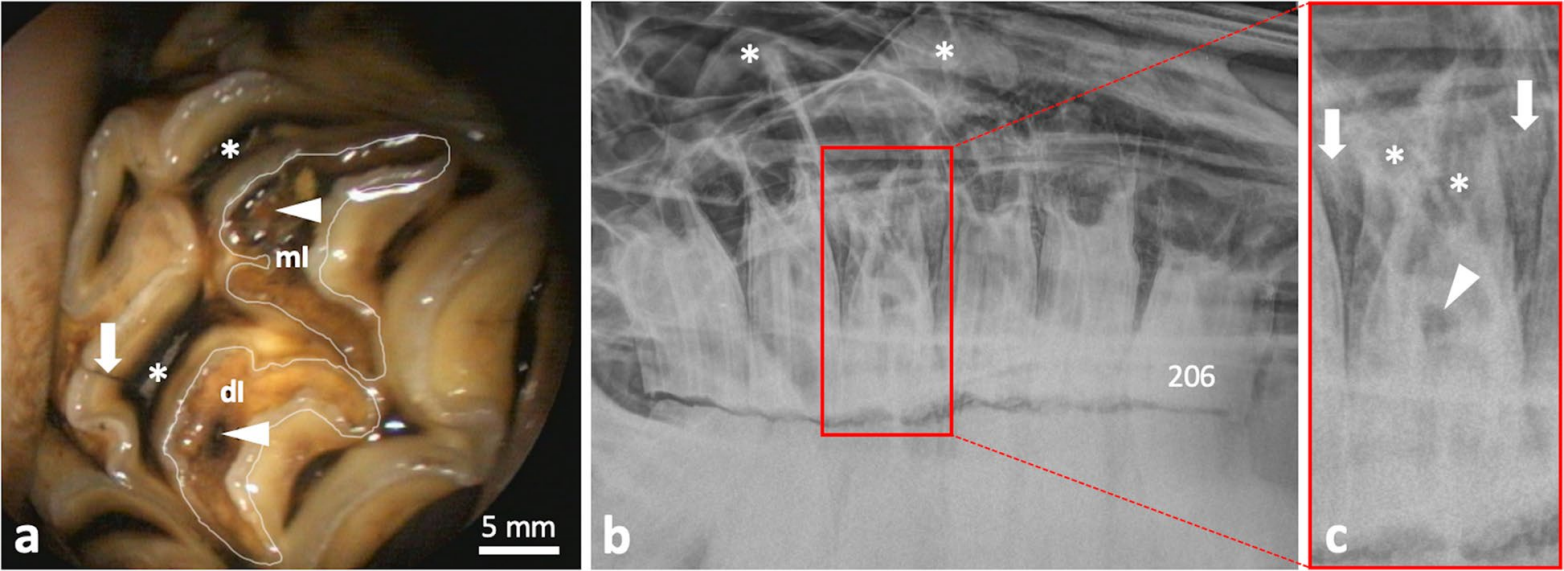
Occlusal abnormalities and radiographic findings.** a** Endoscopic intraoral view on the occlusal surface of the upper left cheek tooth 209. White lines highlight the mesial (mI) and distal infundibulum (dI) along occlusal infundibular enamel folds. Normal colour appearance of dI and darkly discolored mesial infundibular cement of mI indicative for infundibular caries grade 1/(0–4). Both infundibula show a small occlusal opening in cementum (arrowheads) representing a developmental remnant of occlusal blood supply. Pulp horn 3 (upper asterisk) and 4 (lower asterisk) show occlusal dentinal defects. A fissure fracture line extends from peripheral enamel to dentine at pulp position 4 (arrow). **b** Dental radiograph showing left upper cheek teeth 206-211 and not readily assigned soft tissue dense structures (asterisks). **c** Enlarged view of tooth 209 showing radiolucency in the area of the mI (arrowhead) and apical tooth substance (asterisks), as well as periapical/ interalveolar bone sclerosis and thickening (arrows)

Examination of orodental structures was performed in the standing sedated horse using 0.01 mg*kg^−1^ detomidine hydrochloride (Detogesic; Zoetis, Ismaning, Germany) administered intravenously. An oral speculum (Type Hausmann Serie 4000; Equus Dental Harmony, Rochetaillée-sur-Saone, France), 44 cm intraoral endoscope (α = 70° viewing angle; Equine Dental Scope and Tele Pack Vet x LED; Carl Storz, Tuttlingen, Germany), pulp probe, and periodontometer (Pferdefit-Dental, Hohenstein-Breithardt, Germany) were used to assess the oral cavity and to investigate single teeth. Cheek tooth arcades exhibited minor wave-like irregularities. The upper cheek tooth 209 displayed multiple occlusal abnormalities (Fig. 1a). Infundibular caries lesions were assessed according to the modified Honma’s grading system [35]. The gingiva and other oral soft tissues showed no pathological findings.

#### Dental radiography

Radiographs were obtained using a digital radiography (DR) system (IP Cassette Type CC plates and CR-IR 391 RU developer; Fujifilm, Tokyo, Japan). The x-ray beam was directed α = 90°/ +35° according to the hemisphere model recommended by Stoll *et al*. (2011) [36]. Excepting the left upper cheek tooth 209 that showed infundibular radiolucency as well as apical/ periapical bone sclerosis and thickening (Fig. 1b, c), other cheek teeth displayed typical age-related changes, i.e. regular lamina dura and normal appearing interradicular and interdental alveolar bone. Areas of superimposed paranasal sinuses and nasal cavity exhibited abnormal soft tissue dense opacification (Fig. 1b).

#### Rhinoscopy and sinoscopy

**Fig. 2:**
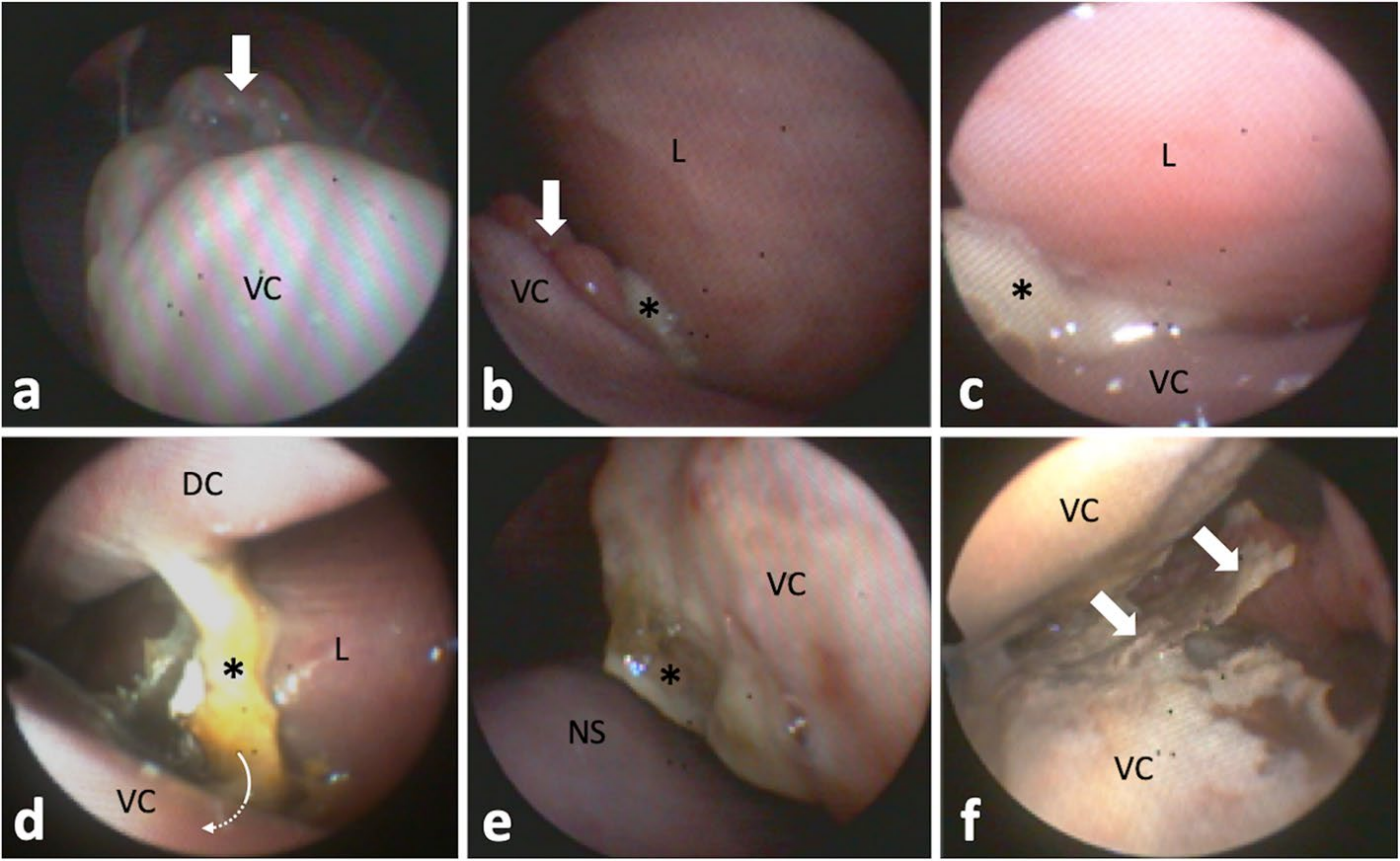
Left sinonasal endoscopic views.** a** Polypoid mucosal swelling (arrow) of caudodorsomedial ventral conchal (VC) aspects. Endoscope is located in the middle nasal meatus laying dorsal on the VC looking caudal. **b** Polypoid mucosal swelling (arrow) of dorsolateral VC aspects near the rostral nasomaxillary aperture, which shows draining purulent secretions (asterisk). The endoscope was guided towards the left lateral wall of the nasal cavity (L). **c** Advancing the endoscope further into the aperture clarified origin of purulent secretions (asterisk) from the rostral maxillary sinus, which was not accessible due to mucosal swelling. **d** At the level of entrance to the VC recess (arrow), masses of isolated necrotic conchal bone and impacted pus (asterisk) are visible. The VC recess was not accessible endoscopically. Dorsal concha, DC. **e** Caudomedial aspects of the deformed VC showing inflamed, partially necrotic, areas with purulent deposits (asterisk). Nasal septum, NS. **f** VC sinus reached via a large necrotic defect in middle medial aspect of the VC. Isolated necrotic conchal bone an pus (arrows) hindered passage into the rostral maxillary sinus

Ensuing transnasal endoscopy in the standing sedated horse using a modified flexible 5 mm human bronchoscope (BF Type P 20; Olympus, Hamburg, Germany) revealed broad pathological changes of left sinonasal structures, including moderate to marked, focal polypoid, mucosal swellings, purulent secretions, and necrotic conchal residues (Fig. 2). The left rostral sinus compartment, i.e. ventral conchal sinus and rostral maxillary sinus, and conchal recesses were not accessible. All other sinonasal structures appeared without discernible changes.

#### Computed tomography of the head

**Fig. 3:**
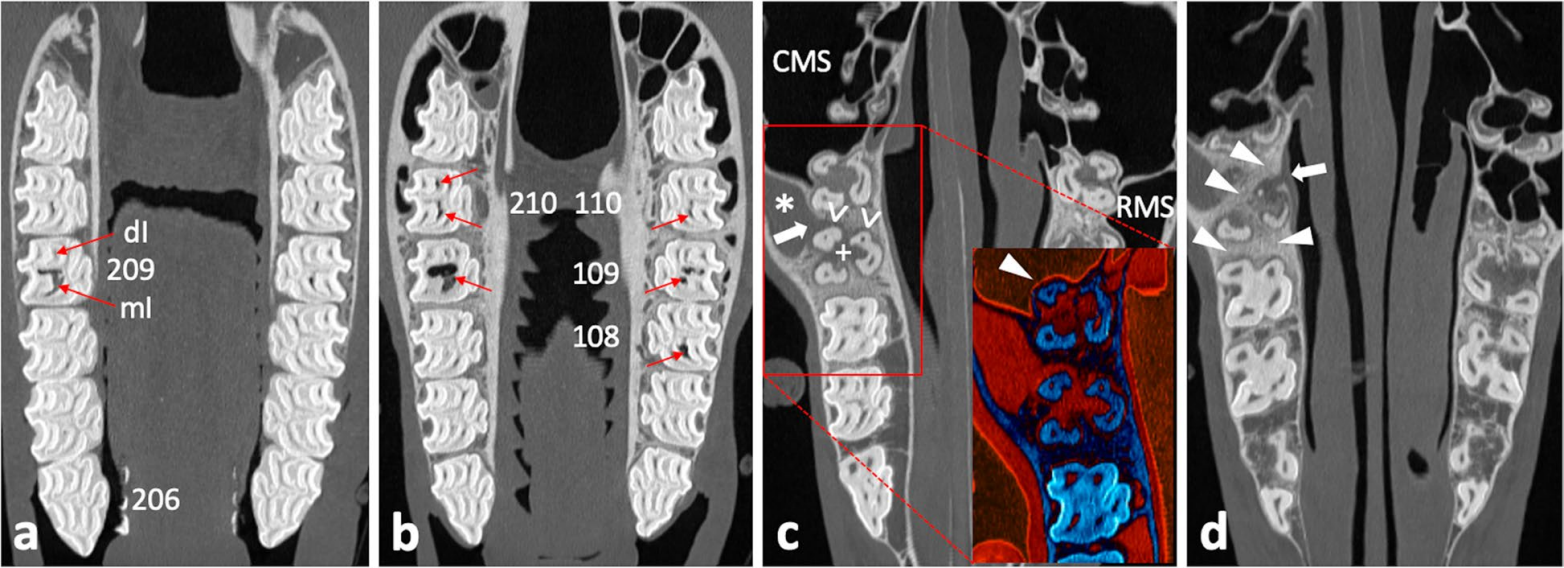
Coronal CT image sections of maxillary cheek teeth and adjacent sinonasal structures. a – d Section plane moving in apical direction, starting 10 mm subocclusal (tooth 209); 0.6 mm slice thickness; W3100/C500. **a** Subocclusal enlargement and filling with hypodense material of the mesial infundibulum (mI) of 209; the distal infundibulum (dI) appears unaltered. **b** At the infundibular fundus level, mI 209 appears highly enlarged and filled with hypodense material and gas; dI appears unaltered. The respective mI of 108, 109, 110, and both infundibula of 210 show accentuated signs of cement hypoplasia and hypodense/ gas filling. **c** Normal pneumatization of caudal maxillary sinuses (CMS) and right rostral maxillary sinus (RMS); soft tissue/ fluid dense filling of left RMS (asterisk). Insert: enhanced visualization of thin sinusoidal mucosal lining at the air interface and normal appearing delicate sinusoidal lamina dura of the apically unaltered neighboring tooth 210 (arrowhead); Perfusion Color Look Up Table (CLUT), soft tissue = red, bone = dark blue. Osteolytic loss of sinusoidal lamina dura of the buccodistal root of 209 resulting in an apicosinusoidal fistula (arrow). Partial loss of interradicular bone (plus) and widening of periapical periodontal space (open arrowheads). **d** Severe sclerosis of interalveolar and periapical alveolar bone of 209 (arrowheads). Inflammation caused discontinuity in the nasal lamina dura of the palatal root, thus apiconasal fistulation (arrow)

**Fig. 4:**
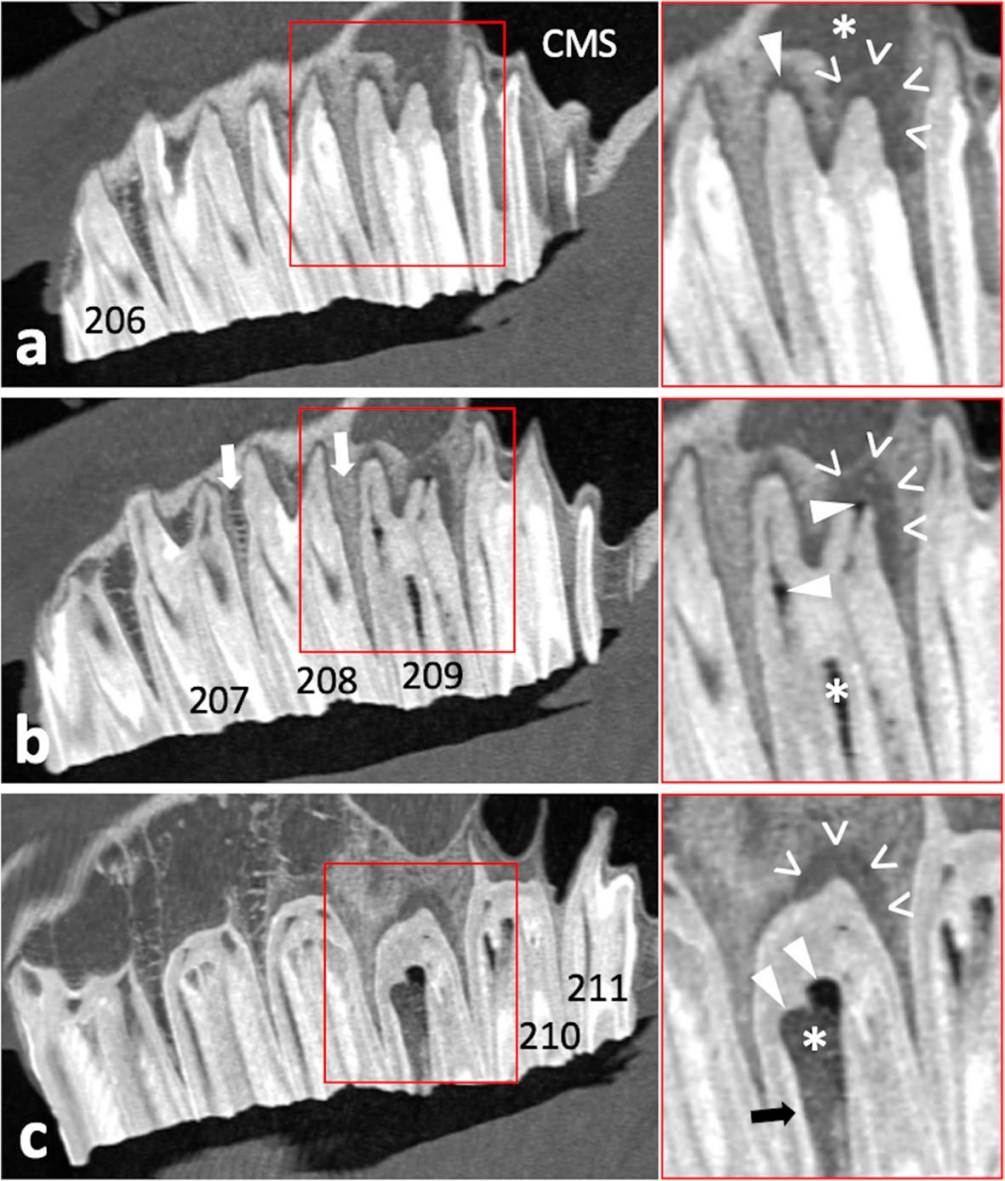
Sagittal CT image sections of left maxillary cheek teeth and adjacent sinusoidal structures. a – c Section plane moving from buccal to medial; 0.6 mm slice thickness; W3100/C500. **a** Osteolytic loss of sinusoidal lamina dura of the buccodistal root of 209 (open arrowheads) and resulting apicosinusoidal fistulation with rostral maxillary sinus showing soft tissue/ fluid dense filling (asterisk). Normal pneumatized caudal maxillary sinus (CMS). Widening of periodontal space buccomesial root of 209 (arrowhead). **b** Gas inclusions in the buccomesial pulp and apical end of the buccodistal pulp (arrowheads). The periodontium at the buccodistal root of 209 shows severe periapical bone loss and inflammatory enlargement of periodontal space (open arrowheads). Slight central infundibular cement loss of the mesial infundibulum 209 (asterisk). Normal trabecular appearance of interalveolar bone between 207/208 and sclerosis between 208/209 (arrows). **c** Subocclusally, the severely enlarged mesial infundibulum of 209 shows missing infundibular cement (hypoplasia) and filling with heterogenic hypodense material and gas (asterisk). Missing enamel lining of the infundibular fundus (arrowheads), while e.g. parietal infundibular enamel is still present (arrow). Severe periapical bone loss and enlargement of the periodontal space (open arrowheads)

**Fig. 5:**
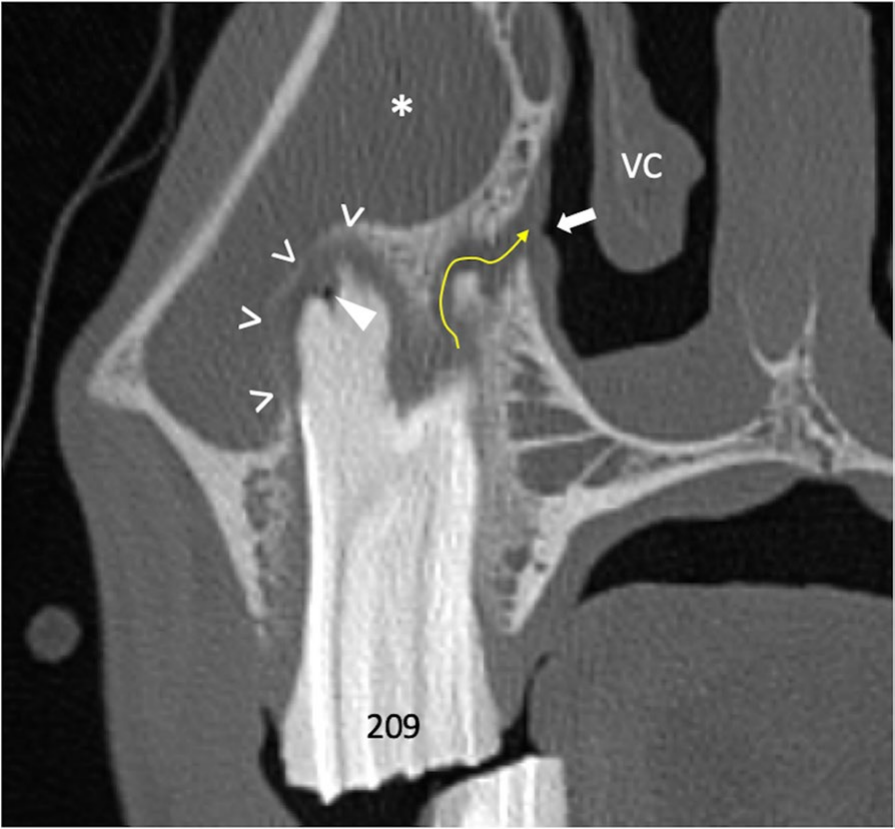
Transversal CT image section of maxillary cheek tooth 209 and adjacent sinonasal structures. 0.6mm slice thickness; W3100/C500. Buccodistal root of 209 showing severe periapical bone loss and enlarged periodontal space (open arrowheads) as well as apical gas inclusion (arrowhead). Filling of the rostral maxillary sinus (asterisk). Apiconasal fistula tract (arrow path) and accompanying swelling of the nasal mucosa (arrow). The ventral concha (VC) appears deformed

**Fig. 6:**
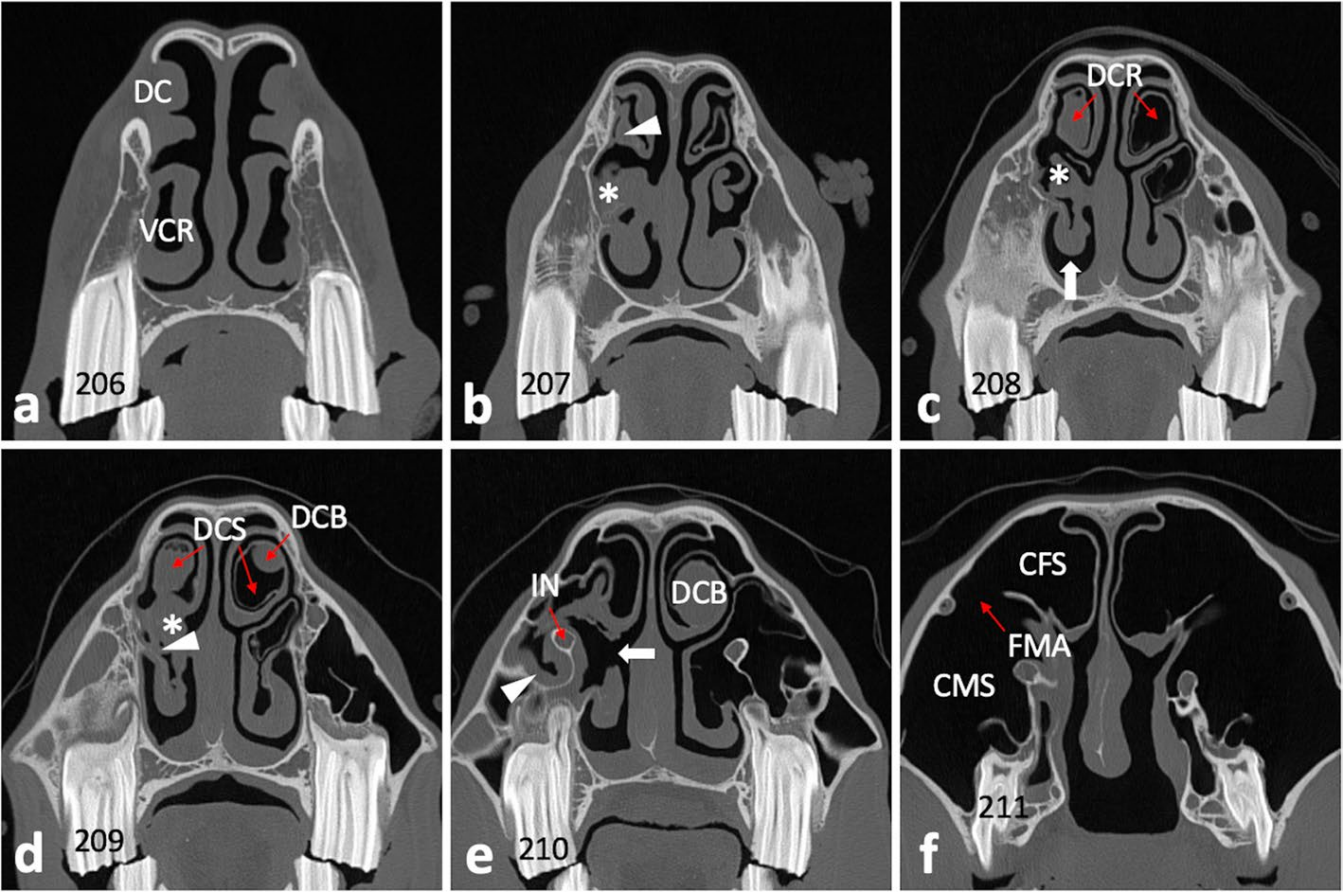
Transversal CT image sections of the head. **a**–**f** Section plane moving from rostral to caudal; 0.6 mm slice thickness; W3100/C500. **a** Dorsal concha (DC) and ventral concha with its ventral conchal recess (VCR) appear unaltered at the level of 106/206. **b** Ventral conchal deformation and disintegrated VCR entrance area (asterisk) at the level of interdental space 07/08. Soft tissue/ fluid dense filling of left dorsal conchal recess (arrowhead). **c** Ventral conchal deformation (asterisk) and retraction (arrow), and filling of the left dorsal conchal recess (DCR) at the level of IDS 08/09. Right DCR appears pneumatized (normal). **d** Soft tissue/ fluid dense filling of the left ventral conchal sinus (asterisk) and VCR (arrowhead), dorsal conchal bullae (DCB), and left dorsal conchal sinus (DCS) at the level of 109/209. **e** At the level of IDS 10/11 the medial aspect of the ventral concha appears with a large necrotic defect (arrow). The mucosa of the rostral maxillary sinus (arrowhead) and around the infraorbital nerve (IN) appears swollen. **f** At the level of 111/211 the caudal maxillary sinus (CMS) and conchofrontal sinus (CFS), which communicate via the frontomaxillary aperture (FMA), appear unaltered

Standing computed tomography (CT) of the head using a big bore sliding gantry (Somatom Sensation Open; Siemens Healthcare, Forchheim, Germany) was performed as previously described [37, 38]. The radiological assessment, using Syngo imaging software (Siemens Healthcare), comprised three-dimensional volume rendering, multiplanar reconstructions, and orthogonal reconstructions. The tooth 209, which showed abnormalities orally and radiographically, exhibited distinct endodontic and periapical affection with apicosinusoidal and -nasal communication, which led to extensive inflammation of the rostral sinus compartment and nasal structures, respectively. The central apical radiolucency on the radiograph turned out to be an extensive subocclusal infundibular caries lesion. For detailed visualization and description of detected pathologies see Figs. 3, 4, 5 and 6.

### Working diagnosis

Infundibular caries grade 2–3/(0–4) of the mesial infundibulum of the cheek tooth 209; pulpitis and apical-/ periapical infection 209 with associated sinusitis of the left rostral maxillary and ventral conchal sinus accompanied by necrosis of the left ventral nasal concha.

### Treatment

Albeit promising reports on orthograde endodontic treatment [39] and infundibular caries restoration [40] in horses, tooth extraction and endoscopic treatment of affected sinonasal structures was recommended, as this is still the most commonly used procedure due to limitations of conservative treatment options. The decision was supported by (i) advanced age of the horse, (ii) profound apical/ periapical affection, (iii) deep infundibular caries that was suggested to have intruded dentinal tubules, (iv) reactive pulp, and (v) extensive sinonasal communication with associated inflammation.

A 130 mm 12-gauge venous catheter (Mila International Inc., Kentucky, USA) was placed in the left jugular vein. The horse received preemptive analgesic treatment with intravenous flunixin meglumine (1.1 mg*kg^−1^; Equibos; Serumwerk Bernburg, Bernburg, Germany). Sedation was performed using a detomidine hydrochloride (0.01 mg*kg^−1^; Detogesic; Zoetis) and butorphanol^12^ (0.01 mg*kg^−1^; Torbugesic; Zoetis) bolus followed by continuous perfusion sedation maintenance (both 0.01 mg*kg^−1^*h^−1^ detomidine hydrochloride and butorphanol) in combination with infusion of 25 mL*min^−1^ Ringers solution (B. Braun, Melsungen, Germany). A left maxillary peripheral nerve block using 12 mL of 2 % lidocaine hydrochloride (bela-pharm, Vechta, Germany) and peridental infiltration anesthesia (gingiva and periodontal space of tooth 209) using 4 mL of 3 % mepivacaine hydrochloride (Scandonest; Septodont, Niederkassel, Germany) were performed.

**Fig. 7:**
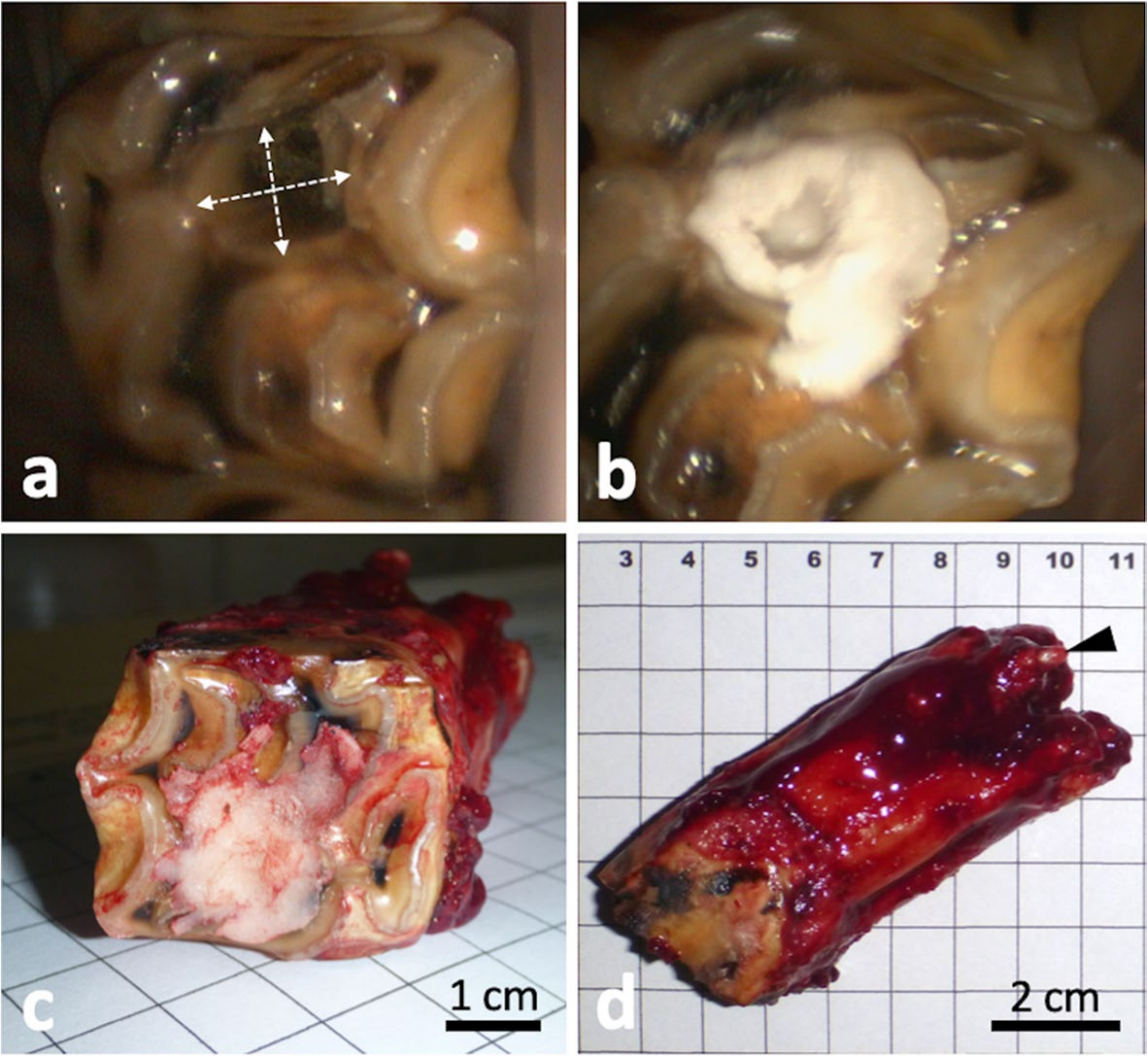
Tooth preparation and postextraction appearance. **a** Orthograde revision of the altered mesial infundibulum and **b**, filling and compaction with PMMA. **c–d** Extracted tooth 209; arrowhead indicating remnants of the apiconasal fistula

The tooth 209 was extracted orally in the standing sedated horse following mechanical debridement and filling of the spacious mesial infundibular caries lesion with thermoplastic polymethylmethacrylate (PMMA; Demotec; Demotec Demel, Nidderau, Germany) (Fig. 7). Prevalence of extraction-related tooth fractures is higher in teeth with large hollow infundibular lesions [41]. We therefore decided to mechanically stabilize the tooth by filling the affected infundibulum prior to extraction. The tooth was carefully removed from the oral cavity avoiding contact with other structures. A sterile swab sample was taken from the inflamed tissue in the trifurcation area of the tooth roots for bacteriological investigation. A postoperative x-ray image showed no dental remnants in the alveolus, which clinically displayed a 3 mm palatal oronasal fistula. Necrotic tissue in the alveolus was removed and the fistula tract was gently revised, both under endoscope guidance. The alveolus was temporarily closed with iodoform gauze (Jodotamp; Nobamed, Wetter, Germany).

Areas including the ventral and middle nasal meatus, nasomaxillary aperture, ventral conchal recess, and sinus system were transendoscopically flushed with water. Due to mucosal swelling and thus impassable rostral nasomaxillary aperture both the rostral maxillary sinus and ventral conchal sinus were reached via the necrotic defect in the medial conchal lining. The rostral maxillary sinus could be indirectly flushed via the conchomaxillary aperture. Inspissated pus and demarked necrotic conchal bone fragments were removed using an 2 mm endoscopy forceps (Fujifilm medwork, Höchstadt/Aisch, Germany); no bacteriology was done on sinus secretions and removed necrotic tissue.

### Bacteriology

**Fig. 8:**
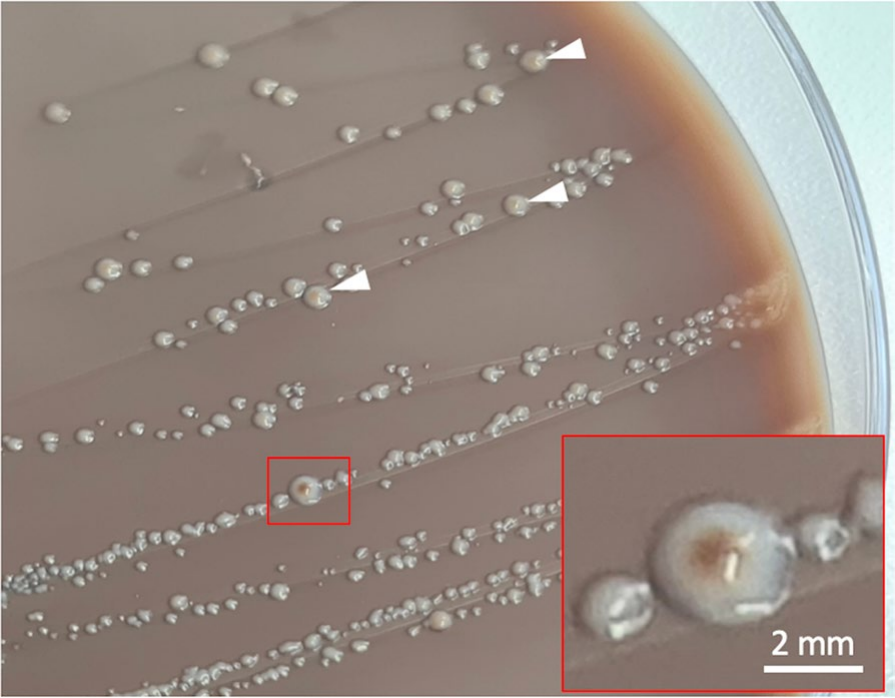
*Prevotella histicola* colony morphology on Schaedler KV agar with 5 % sheep blood. Bull’s-eye like pigmentation of single colonies (insert) after 72 h anaerobic incubation. Some other colonies with incipient pigmentation (arrowheads)

Gram-staining of the swab sample material revealed a high number of primarily Gram-negative rods and erythrocytes. The swab sample material was inoculated using following agar plates (Becton Dickinson, New Jersey, USA): (i) for isolation of aerobic bacteria on Columbia agar with 5 % sheep blood, Columbia colistin nalidixic acid (CNA) agar with 5 % sheep blood, MacConkey-II agar, and Mueller-Hinton chocolate agar (incubation: 24 h at 36 ± 1 °C with 5 % CO_2_ and 15–17 % O_2_); (ii) for nonselective isolation of anaerobic bacteria on Schaedler agar with vitamin K1 and 5 % sheep blood and Schaedler kanamycin vancomycin (KV) agar with 5 % sheep blood (incubation: 72 h at 36 ± 1 °C in a CO_2_ enriched anaerobic environment); and (iii) for selective isolation of Gram-negative obligate anaerobic rods, particularly *Prevotella *ssp*.* and *Bacteroides* ssp., on Schaedler KV agar with 5 % sheep blood (incubation: 7 days at 36 ± 1 °C in a CO_2_ enriched anaerobic environment). The growth pattern and colony morphologies were compared between plates incubated under either aerobic or anaerobic conditions. Aerobic incubation showed no growth of pathogens, but little growth of bacteria of the normal mucosal flora. Anaerobic incubation revealed extensive growth of predominantly Gram-negative rods that are suspected to be the main pathogenic microorganism from the sample material; first assessment after 72 h. Single colonies morphologically appeared entire circular, convex, cream colored, and opaque, showing a diameter of up to 2 mm. After 72 h of incubation, many colonies developed a reddish-brown pigmented center (bull’s-eye appearance), whereas others exhibited no pigmentation (Fig. 8). The relative number of pigmented colonies increased until day 7.

Suspected colonies were further differentiated by bacterial fingerprint identification using Bruker Microflex LT matrix-assisted laser desorption ionization time-of-flight mass spectrometry (MALDI-TOF MS) connected to Bruker FlexControl (version 3.4) and Bruker CompassFlex Series (version 1.4) software, and Bruker general reference library (Bruker Daltonik, Bremen, Germany). Sample preparation, measurement, and identification followed manufacturer information. According to Nagy *et al*. (2016), a (log) identification score ≥ 2.0 was assumed representing identification at species-level, 1.7–1.99 at genus-level, and < 1.7 indicates non-reliable identification [42]. Mass spectra from repeated measures of unpigmented and pigmented anaerobic colonies (after 72 h and 7 days as a control) confirmed isolation of the Gram-negative rod-shaped bacterium *P. histicola* (all log scores > 2.0).

### Postoperative management

The horse received postoperative antiphlogistic treatment with flunixin meglumine (0.55 mg*kg^−1^ p.o. BID; Finadyne; MSD Animal Health, Unterschleißheim, Germany) for five days, secretolytic treatment with acetylcysteine (1 g*100 kg^−1^ p.o. BID; Equimucin; CP-Pharma, Burgdorf, Germany) for 24 days, and initial antibiotic treatment with sulfadimethoxine trimethoprim (30 mg*kg^−1^ p.o. BID; Medistar, Ascheberg, Germany). After 4 days, antibiotic treatment was adapted according to the results of bacterial investigation. Anaerobe antibiotic treatment is predominantly based on empirical value, thus literature evidence on *Prevotella *ssp*.* susceptibility was considered [32, 43–48], and antibiotic treatment changed to metronidazole (20 mg*kg^−1^ p.o. TID; Metrobactin; CP-Pharma, Burgdorf, Germany) for 12 days.

**Fig. 9:**
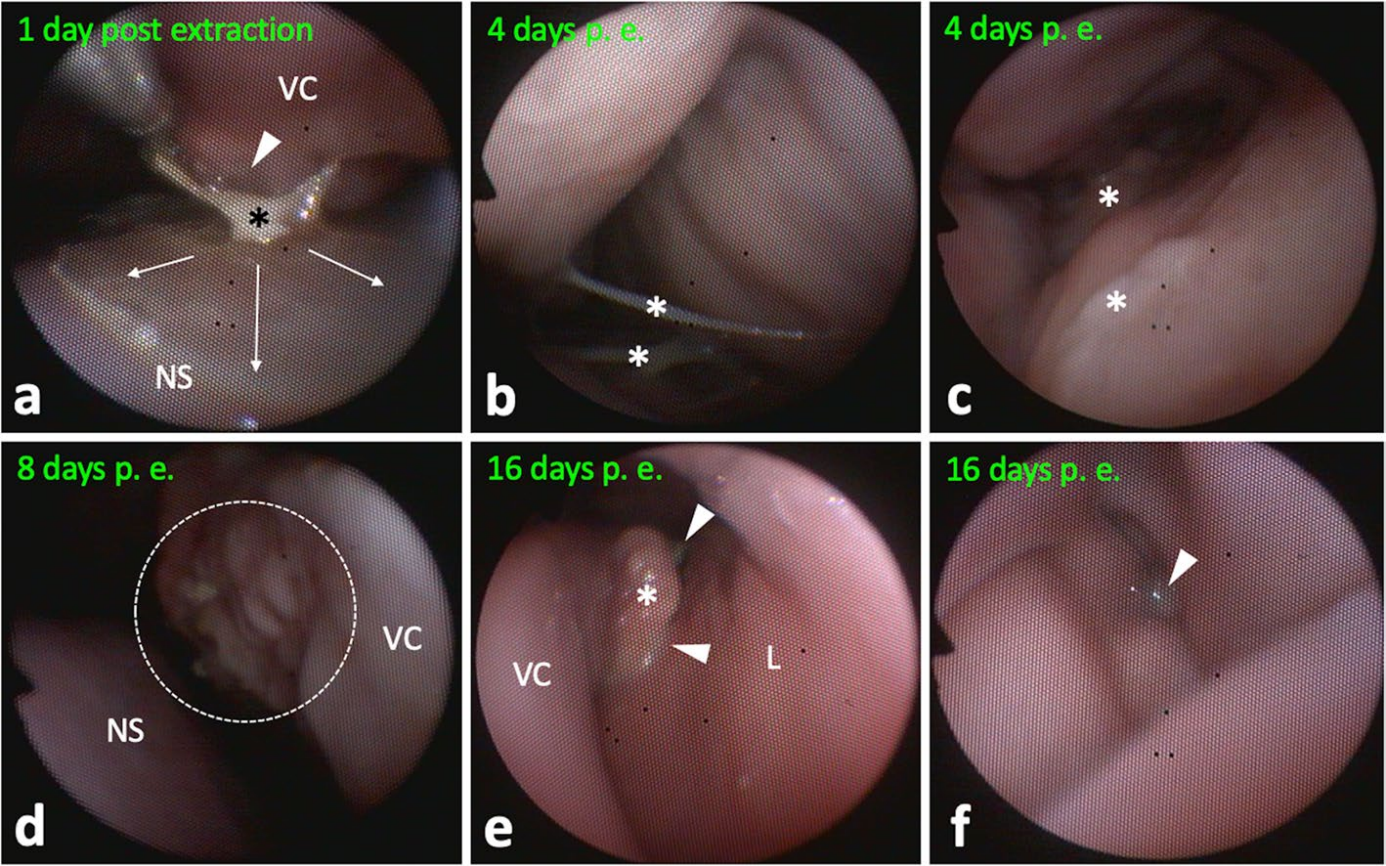
Left sinonasal endoscopic views of continuous follow up examinations. **a** Removal of a conchal bone sequestrum (asterisk) at the medial aspect of the ventral concha (VC) using endoscopic forceps. The subjacent mucosa shows some irregularities (arrowhead), which may be sign of early granulation. Arrows indicate focal purulent membranous deposits on the nasal septum (NS). **b** Mucopurulent secretions (asterisks) in the rostral entrance part to the ventral conchal recess and **c**, in the caudal ventral conchal recess. **d** Former severely necrotic medial aspect of the VC exhibiting granulation (dotted rim). **e** Area of the rostral nasomaxillary aperture showing little seromucous secretion deposit (arrowheads) around a polypoid VC deformation (asterisk). L, lateral (maxillary) mucosa. **f** Small amounts of seromucous secretion (arrowhead) in the caudal ventral conchal sinus

The alveolus was first inspected 4 days post extraction under sedation with intravenous xylazine hydrochlorid (0.5 mg*kg^−1^; Xylavet; CP-Pharma). The area of the debrided fistula was already closed by a stable blood clot. After gentle flushing the alveolus, a iodoform gauze was again used as an alveolar plug. The alveolus was further checked at 6-day intervals until discharge of the horse at day 16 post extraction. On the day of discharge, the alveolus showed good granulation and the fistula remained closed. Affected sinonasal structures were checked under xylazine sedation and abundantly flushed transendoscopically at day 1, 4, 8, and 16 post extraction. Necrotic tissue, sequestrated conchal bone and mucopurulent secretions were removed to the greatest possible extent. Five days after initial treatment, malodor from the nostrils diminished. Endoscopic examination 8 days post extraction showed little seromucous secretion and no necrotic conchal remains. An endoscopic follow up can be seen in Fig. 9. Mild unilateral seromucous nasal secretion was still present on the day of discharge.

### Long-term follow up

One week after patient discharge, a follow up phone call provided information that the mild seromucous secretion had ceased. After 18 months, the owner reported that there had been no problems in the meantime and that regular checks by the private veterinarian were normal.

## Discussion and conclusions

This report presents the first documented case of apical/ periapical tooth infection and accompanying sinonasal changes caused by the facultative human oral pathogen *P. histicola* in the horse. The affected upper cheek tooth 209 exhibited extensive subocclusal cemental hypoplasia and caries of the mesial infundibulum, an occlusal fissure fracture, and signs of endodontic inflammation. A multimodal treatment approach, including intraoral exodontia, transendoscopic removal and lavage of altered sinonasal structures, and targeted antibiotic treatment resulted in healing without short and midterm complications.

Observed colony morphologies and variable pigmentation of isolated anaerobes coincide with the original *P. histicola* strain description in 2008 [17]. Gram-staining and mass spectrometry substantiate the assumption that *P. histicola* may have caused dental infection in the presented horse. Hitherto, *P. histicola* was only documented in man as an obligate anaerobe, Gram-negative staining bacterium that under certain conditions causes dental pathologies such as caries and periodontitis [16–21]. Nevertheless, yet no obligate pathogenic *Prevotella* species has been described [49]. Although most of the around 50 characterized species in the genus *Prevotella* are linked to humans, also other mammals or the environment can be inhabited [49]. Cross-species distribution seems not unlikely, since e.g. *P. melaninogenica* and *P. oralis* were isolated both from human and equine patients [23, 32, 33]. On a genus-level, *Prevotella* ssp. were also found in human acute odontogenic maxillary sinusitis [50], and in pulpitis, apical infection, periodontal disease, and odontogenic sinusitis in horses as well [10, 22–25].

In the presented case, the mesial infundibulum exhibited cement hypoplasia resulting in a hollow subocclusal mesial infundibular cavity associated with infundibular caries. Due to lack of enamel lining of the infundibular fundus, microorganisms are likely to have invaded subjacent dentinal tubules, causing pulpitis. However, it is not clear whether lack of infundibular fundus enamel lining was due to enamel hypoplasia or the carious process. Dacre *et al*. (2008) reported that in horses 16 % of apical infections in upper cheek teeth are caused by infundibular caries [12]. Windley *et al*. (2009) identified 90 % of investigated infundibula showing at least mild infundibular lesions, but in only 65 % visible changes on the occlusal surface occurred [51]. In a study examining mainly middle-aged working horses, we previously observed a 32.9 % prevalence of cheek tooth occlusal infundibular caries lesions [52]. Similarly, in the presented case, carious occlusal changes of the tooth 209 were less severe compared to the extensive subocclusal process. The large subocclusal infundibular cavity and more severe carious lesions could not be seen orally. The mesial infundibulum showed slightly dark discoloration and both the mesial and distal infundibulum showed a small occlusal opening in the infundibular cementum. These openings, anecdotally described as a pathology, actually are developmental residuals from preeruptive infundibular blood vessels [53]. Nevertheless, connection of occlusal openings with spacious subocclusal infundibular lesions can cause apical food transportation and thus bacterial niche formation. As is the case in the mesial infundibulum of the reported case, Brühler *et al*. (2014) observed bulbous enlargement of the apical infundibular aspect in 3.4 % of the 1176 investigated infundibula [3]. Compared with the distal infundibulum, the prevalence of cemental hypoplasia and caries is higher in the mesial infundibulum [3, 7, 51]. Interestingly, developmental blood supply to the distal infundibulum likely persists longer, thus leading to a more complete cement formation [53]. Advanced infundibular caries may also predispose to iatrogenic tooth fractures during extraction [41]. Therefore, the subocclusal bulbous infundibular lesion was debrided and filled with thermoplastic PMMA before extraction, thus avoiding iatrogenic crown fractures during extraction and subsequent complications.

Acid producing *Streptococcus devriesei*, which is related to *Streptococcus mutans* from human caries, was previously found to be associated with equine infundibular caries lesions [54]. Also *P. histicola* produces acidic metabolites that may cause damage to dental hard substances [17], actually was found in human caries [19, 20]. Yang *et al*. (2012) investigated saliva from humans with active carious lesions and found *Prevotella* to be the only over-abundant bacteria genus [55]. Nevertheless, it remains debatable whether in the presented case anachoresis, bacterial niche formation due to the observed fissure fracture, or the lesion have caused apical infection. Pollaris *et al*. (2020) described deep running occlusal fissure fractures to affect adjacent pulp horns by bacterial niche formation [15]. In the presented case pulp horns # 3 and # 4, which showed CT signs of pulpitis (gas inclusions), were associated with occlusal secondary dentine defects. In 32 % of apically infected cheek teeth occlusal pulp exposure occurs [11]. Such defects in secondary dentine need time to emerge on the occlusal surface, which is driven by continuous tooth eruption in horses. This indicates a chronic bacterial pulpal impact, as it might be the case for infundibular caries or fissure fractures. Although in the presented case both the affected mesial infundibulum and fissure fracture are located closer to pulp horn # 3, pulp horn # 4 was also affected. In equine upper cheek teeth, a large variety of communications between the pulp horns was previously described [56], and thus spread of infection from pulp horn # 3 in other parts of the pulp system is very likely.

Beside communication of apical infection with the rostral maxillary sinus, apiconasal fistulation may have caused spread of anaerobes via the nasal and conchal mucosa to contribute to the extensive conchal necrosis. A limiting factor of this study is that we have not bacteriologically investigated the sinus secretions, nasal necrosis, or carious infundibulum, which may lead to the assumption that isolated *P. histicola* could have originated from swab or sample contamination. However, the tooth was removed carefully from the oral cavity so that the apical area experienced no contact with other oral structures. The inflamed apical trifurcation area, which is protected by the flanking tooth roots, was dabbed deeply in addition. The huge density of Gram-negative rods in the initial Gram-staining and uniform anaerobe cultures further make contamination very unlikely. Results indicate that *P. histicola* was the predominant bacterial species at the infected tooth root apices. Another limiting factor in this study is that the extracted tooth was not examined pathohistologically, which may have helped verifying bacteriological affection.

Gram-negative obligatory anaerobes such as *Prevotella* ssp., *Fusobacterium* ssp., *Porpyhromonas* spp. and *Bacteroides* ssp. were found to be the predominant bacteria genus present in odontogenic sinusitis cases [10, 24, 25, 57]. The occurrence of obligate anaerobes also differs in primary sinusitis cases and healthy samples [57]. It is obvious that *P. histicola* may be able to also colonize this niche in the horse when invading from apical infections. Due to suspected sampling-associated cross-contamination, bacteriological examination of sinusitis cases in our clinic is routinely carried out only if the affected sinus compartment has to be trephined under aseptic conditions; extracted teeth are always sampled at inflamed roots. Transendoscopic catheter-based sampling of inflammatory secretions emerging from the nasomaxillary apertures was currently described to facilitate diagnostics in sinusitis cases in the horse [57], but has not yet been established in routine diagnostics.

In the presented case, no antibiotic resistance testing was performed. The laboratory referred to an usual susceptibility of *Prevotella* ssp. to amoxicillin and clavulanic acid, chloramphenicol, marbofloxacin, enrofloxacin, pradofloxacin, cephalosporines of 3rd and 4th generation, clindamycin, lincomycin and metronidazole. In the past there were no generally accepted methods for testing antimicrobial sensitivity of Gram-negative anaerobes, which is why selection of antibiotics was often empirical [48]. But also nowadays, despite advanced techniques for antibiotic sensitivity testing, difficulties in anaerobe sampling, transport, and *in vitro* cultivation set empirical antibiotic treatment for anaerobe infections over susceptibility testing, which is mostly very time-consuming [58]. However, current evidence suggests that in last decades an increase and dynamic change in antibiotic resistance in *Prevotella* ssp. occured [46]. *Prevotella* ssp. show resistance against amoxicillin, carbapenems, clindamycin [43, 47]. An increasingly high (~ 95 %) resistance against penicillin and ampicillin [46, 58] may have contributed to persistence of symptoms despite penicillin treatment prior to referral in the presented case. Metronidazole is widely used to treat infections of the oral cavity and other infections with anaerobic microorganisms in humans [48]. Nevertheless, resistances are increasing here too, but susceptibility of *Prevotella* ssp. to metronidazole is still up to 100 % [32, 43–47]. Thus, we decided to use metronidazole. Importantly, in the EU the horse is then no longer suitable for human consumption, which must be noted in the equine identification document. In humans, duration of treatment is normally recommended for 14 days [48], however, there are no data reporting optimum duration of treatment in horses. In the current case 12 days of administration was chosen, as this was within the time span the horse stayed hospitalized. In a case of medial pterygoid myositis caused by *Prevotella* ssp. in a horse, metronidazole was discontinued five days after surgery but infection reemerged within one month post-surgery [32]. This might indicate that in horses, metronidazole should be administered at least longer than 5 days and that anaerobes likely recolonize affected areas when treated improperly.

The presented report provides the first documented case of apical/ periapical infection with accompanying sinonasal affection in a horse caused by *P. histicola*, a human oral commensal and facultative pathogen. This case further indicates human-horse cross-species distribution of *P. histicola*, at once highlights the necessity for more sufficient microbiological diagnostics and targeted antibiotic treatment in equine dental practice.

## Data Availability

All data generated and analyzed during this study are included in this published article.

## Abbreviations

BID: *bis in die* (two times a day)
CNA: Colistin nalidixic acid
CT: Computed tomography
DR: Digital radiography
KV: Kanamycin vancomycin
MALDI-TOF MS: Matrix-assisted laser desorption ionization time-of-flight mass spectrometry
PMMA: Polymethylmethacrylate
TID: *ter in die* (three times a day)
